# Tropisetron Protects Against Acetaminophen-Induced Liver Injury via Suppressing Hepatic Oxidative Stress and Modulating the Activation of JNK/ERK MAPK Pathways

**DOI:** 10.1155/2016/1952947

**Published:** 2016-11-07

**Authors:** Fu-Chao Liu, Hung-Chen Lee, Chia-Chih Liao, Allen H. Li, Huang-Ping Yu

**Affiliations:** Department of Anesthesiology, Linkou Chang Gung Memorial Hospital and College of Medicine, Chang Gung University, Taoyuan 33305, Taiwan

## Abstract

*Objectives*. To investigate the protective effects of tropisetron on acetaminophen- (APAP-) induced liver injury in a mice model.* Methods*. C57BL/6 male mice were given tropisetron (0.3 to 10 mg/kg) 30 minutes before a hepatotoxic dose of acetaminophen (300 mg/kg) intraperitoneally. Twenty hours after APAP intoxication, sera alanine aminotransferase (ALT) and aspartate aminotransferase (AST) levels, hepatic myeloperoxidase (MPO), malondialdehyde (MDA), glutathione (GSH), and superoxide dismutase (SOD) activities, and liver histopathological changes were examined. The MAP kinases were also detected by western blotting.* Results*. Our results showed that tropisetron pretreatment significantly attenuated the acute elevations of the liver enzyme ALT level, hepatic MPO activity, and hepatocytes necrosis in a dose-dependent manner (0.3–10 mg/kg) in APAP-induced hepatotoxicity mice. Tropisetron (1 and 3 mg/kg) suppressed APAP-induced hepatic lipid peroxidation expression and alleviated GSH and SOD depletion. Administration of tropisetron also attenuated the phosphorylation of c-Jun-NH2-terminal protein kinase (JNK) and extracellular signal-regulated kinase (ERK) caused by APAP.* Conclusion*. Our data demonstrated that tropisetron's hepatoprotective effect was in part correlated with the antioxidant, which were mediated via JNK and ERK pathways on acetaminophen-induced liver injury in mice.

## 1. Introduction

Acetaminophen (*N*-acetyl-para-aminophenol [APAP]) is a widespread and very effective drug used as antipyretic and analgesic [1]. However, APAP overdose can cause severe liver injury and even acute liver failure [2, 3]. The initial event in APAP-induced liver injury is a toxic-metabolic damage depending on the formation of a reactive metabolite, N-acetyl-p-benzoquinone imine (NAPQI), which reacts rapidly with glutathione [3]. If the formation of NAPQI exceeds the capacity of hepatocellular GSH, it will covalently bind to cellular proteins (especially in mitochondria). The mitochondrial protein binding is thought to disturb the mitochondrial respiration, and it enhances reactive oxygen species (ROS) and peroxynitrite formation leading to mitochondrial dysfunction [4, 5]. Increased production of reactive oxygen species caused hepatocyte necrosis and apoptosis. MDA, SOD, and GSH are important markers of oxidative stress. Previous reports showed that suppression of the oxidative stress response could attenuate APAP-induced liver intoxication [2, 6]. Previous evidence indicated that caspase-3 activity involves hepatic apoptosis in liver injury [7]. APAP exposure induced caspase-3 activation and promoted mitochondrial cytochrome* c* release into the hepatic cytosol [8]. In addition, growing evidences showed that liver innate immunity participates in the progression and severity of liver injury. The secondary activation of the innate immune response involves upregulation of inflammatory chemokines and cytokines with activation of immune cells (including neutrophils, macrophages, and T cells). The inflammatory mediators such as chemokines, cytokines, ROS, and nitrogen species released by macrophages/Kupffer cells have also been expressed in APAP hepatotoxicity [9, 10].

Previous evidences indicate that intracellular signaling mitogen-activated protein kinase (MAPK) pathways also play an important role in APAP-induced liver injury. c-Jun N-terminal kinase (JNK) is a member of the MAPK superfamily, in which JNK activation can induce mitochondrial oxidant stress in APAP toxicity. Also, inhibition of APAP-induced JNK activation (phosphorylation) leads to decreased mitochondrial permeabilization and cytochrome* c* release [11]. Previous study [12] also showed that the JNK2 signaling plays a protective role in APAP-induced liver injury mice by modulating hepatocellular proliferation and repair. Furthermore, JNK inhibitor (SP600125) effectively reduced APAP hepatotoxicity, in which the main protective mechanisms are decreasing mitochondrial oxidant stress and peroxynitrite production [13]. Extracellular signal-regulated kinase (ERK) also plays an important role in inflammation and oxidative stress [14]. Previous report showed that acetaminophen-induced ERK activation was found in rat neurons and mice liver [14]. Attenuation of ERK activation can decrease APAP-induce hepatic injury [15].

5-Hydroxytryptamine subtype 3 (5-HT_3_) receptor inhibitors are well known as antiemetics used to improve surgery or chemo/radiation therapies-induced nausea and vomiting. Tropisetron, a 5-HT_3_ receptor antagonist, has been reported to be involved in various cell immune responses [16, 17]. Previous studies indicated that tropisetron possesses antiphlogistic and anti-inflammatory effects, which was thought to be related to the inhibition of proinflammatory mediator release and reduction of oxidative stress [18–21]. Recent reports have shown that tropisetron pretreatment has salutary effects on cisplatin-induced nephrotoxicity [22] and vincristine-induced neurotoxicity [23] in rodent models. Our previous study has also shown that tropisetron treatment can decrease hepatic injury following trauma-hemorrhage [24]. It is implied that tropisetron may exert protective effects in response to drug-induced organ injury. However, it remains unknown whether administration of tropisetron possesses protective effects on APAP-induced liver injury. In this study, we investigated the mechanisms of tropisetron on acetaminophen- (APAP-) induced liver injury in mice.

## 2. Material and Methods

### 2.1. Animal Models

C57BL/6 male mice at 8-9 weeks of age were used for these studies. All strains of mice were maintained in C57BL/6 background for the studies. All animal experiments were performed according to the guidelines of the* Animal Welfare Act* and* The Guide for Care and Use of Laboratory Animals* from the National Institutes of Health. All procedures and protocols were approved by the Institutional Animal Care and Use Committee of Chang Gung Memorial Hospital.

### 2.2. Chemicals

APAP and tropisetron were purchased from Sigma-Aldrich (St. Louis, MO, USA). Detection ELISA kit for GSH, SOD, and MDA was purchased from Cayman (Ann Arbor, MI, USA). All chemicals and solvents used in this study were of analytical grade. APAP was dissolved in warm saline (20 mg/mL) for intraperitoneal injection.

### 2.3. Surgical Technique and Acetaminophen-Induced Hepatotoxicity

All animals were housed in an environmentally controlled room with a 12 h light/dark cycle and allowed free access to food and water. Mice were given tropisetron (0.3 to 10 mg/kg) 30 minutes before a hepatotoxic dose of acetaminophen (300 mg/kg) administered intraperitoneally. Mice were anesthetized by ketamine (100 mg/kg) and xylazine (10 mg/kg) mixture using intraperitoneal injection. Body temperature of animals was monitored by TH-8 Thermalert monitoring thermometer (Physitemp) and maintained at 35°C by TCAT-1A temperature control. The samples of this group of mice were collected 20 hours after acetaminophen-induced liver injury and submitted to analysis.

### 2.4. Blood Collection

Blood was collected using a 22-G needle containing anticoagulant by cardiac puncture of anesthetized mice. 500 *μ*L blood was taken into a 1.5 mL tube containing 20 U/mL of heparin/TBS. Blood was maintained at room temperature (RT) before analysis for 2-3 hours.

### 2.5. Serum Assay

Blood was collected at 20 h after APAP administration. Sera ALT and AST levels were measured by VITROS ALT DT and AST DT Slides of the VITROS DT60 II Chemistry System.

### 2.6. GSH and SOD Activities and Hepatic Lipid Peroxidation Assay

The liver tissue sample was homogenized on ice with 9 volumes of pH 7.4 cold Tris-HCl buffer (containing 0.1 mM EDTA-2Na, 10 mM sucrose, and 0.8% saline) [14]. The homogenate was centrifuged at 4°C (10,000 g, 15 min) and the supernatant was used for the determination of GSH, SOD, and MDA following the manufacturer's instructions (Cayman Chemical Co., Ann Arbor, MI, USA). Protein concentration was determined using a Pierce BCA Protein Assay Reagent Kit (Bio-Rad, USA). The GSH activity was detected through yellow 5,5′-dithiobis (2-nitrobenzoic acid). SOD activity was detected utilizing the nitroblue tetrazolium staining method. The MDA was measured through the thiobarbituric acid-reactive substances assay.

### 2.7. Tissue MPO

Mouse livers were submerged in 10 volumes of ice-cold 50 mM KPO_4_ buffer, pH 7.4, and homogenized with a Tekmar tissue grinder. The homogenate was centrifuged at 15,000 g for 15 min at 4°C, and the supernatant was discarded. The pellet was washed twice, resuspended in 10 volumes of ice-cold 50 mM KPO_4_ buffer, pH 6.0, with 0.5% hexadecyltrimethylammonium bromide (Fluka), incubated at 60°C for 2 hr, and then sonicated for 10 sec. The suspension was subjected to three freeze/thaw cycles. Then samples were sonicated for 10 sec and centrifuged at 15,000 g for 15 min at 4°C. Supernatant was added to an equal volume of a solution consisting of ө-dianisidine (10 mg/mL) (Sigma), 0.3% H_2_O_2_ (Sigma), and 50 mM KPO_4_, pH 6.0. Absorbance was measured at 460 nm over a period of 5 min.

### 2.8. HE Stain

The left liver lobes were cut out and fixed in 10% formalin solution. Formalin-fixed tissue samples were embedded in paraffin and 4 *μ*m sections were cut. Replicate sections were stained with hematoxylin and eosin (H&E) for blinded evaluation of the areas of necrosis by the pathologist. The percent of necrosis was estimated by evaluating the number of microscopic fields with necrosis compared to the entire cross section.

### 2.9. Western Blotting Analysis

The total protein extracts were made by pulverization in a grinder with liquid nitrogen, then using a ratio of 1 mL lysis buffer (150 mmol/L NaCl, 1.0% NP-40, 0.5% NaVO_4_, 0.1% SDS, and 50 mmol/L Tris, pH 7.5) containing 1 mmol/L PMSF for each 100 mg of powdered liver sample. Liver lysates (40 *μ*g) were electroblotted onto a PVDF membrane following separation on 8%–12% SDS polyacrylamide gel electrophoresis. Blotted membranes were blocked with 5% skim milk in incubation buffer at room temperature, followed by incubation overnight at 4°C with 1 : 1000 dilution of JNK and ERK (Cell Signaling Technology, MA, USA), and phospho-JNK, phospho-ERK (Cell Signaling Technology, MA, USA) primary antibody. Bound antibody was detected with horseradish peroxidase- (HRP-) conjugated secondary antibody (Cell Signaling Technology, MA, USA) and immunodetected proteins were visualized using the ECL system. Loading accuracy was evaluated by membrane rehybridization with monoclonal antibodies against *β*-actin (Sigma, St. Louis, MO, USA).

### 2.10. Statistical Analysis

All values were expressed as mean ± SEM (*n* = 6 mice/group). All results were analyzed using one-way analysis of variance (ANOVA) and Tukey's multiple comparison tests. Survival was analyzed by Fisher's exact test (*n* = 8 mice/group). Statistically significant differences between groups were defined as a *P* value of ≤0.05. Calculations were performed with the* GraphPad Prism 4.0 Software* (GraphPad Software Inc., San Diego, USA). The statistical methods of this study were reviewed by Clinical Informatics and Medical Statistics Research Center of Chang Gung Memorial Hospital.

## 3. Results

### 3.1. Effect of Tropisetron on Sera ALT and AST Levels

**Figure 1:**
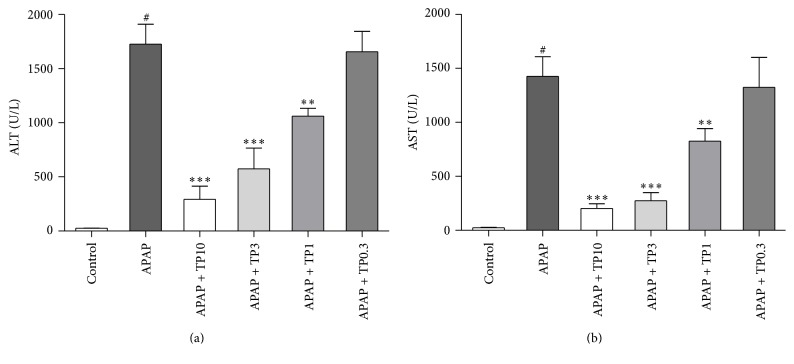
Protection effect of tropisetron in APAP-induced liver injury. Mice were intraperitoneally administered with APAP 300 mg/kg alone or with various concentrations of tropisetron (TP: 0.3 to 10 mg/kg) at 30 min before APAP injection and were sacrificed 20 h after administration for assay of sera ALT (a) and AST (b). Results are mean ± SEM; *n* = 6 mice per group. ^#^ *P* < 0.005 versus control (normal saline); ^*∗∗*^ *P* < 0.01; ^*∗∗∗*^ *P* < 0.005 versus APAP alone.

Serum levels of liver enzymes ALT and AST are shown in Figure 1. A single dose of APAP (300 mg/kg) markedly increased the sera ALT and AST levels (*P* < 0.05) when compared with the control animals. Pretreatment with tropisetron 30 min prior to APAP administration significantly reduced sera ALT and AST levels. Sera ALT and AST levels were markedly decreased in APAP combination with tropisetron groups (1, 3, and 10 mg/kg) compared with the APAP-only group, respectively. Posttreatment with tropisetron 30 min after administration of APAP was evaluated. The serum ALT levels were significantly decreased in tropisetron (3 mg/kg) pre- and posttreated mice compared with the APAP-only mice. However, there is not a statistical difference between pretreated and posttreated groups with tropisetron (3 mg/kg) (Figure S1 in Supplementary Material available online at http://dx.doi.org/10.1155/2016/1952947).

### 3.2. Effect of Tropisetron on Hepatic MPO Expression

**Figure 2:**
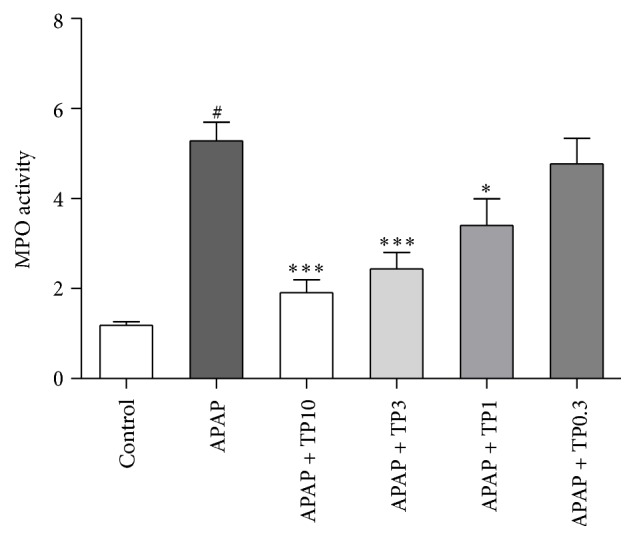
Tropisetron protects against APAP-induced hepatic MPO increase. Injured mice were given APAP 300 mg/kg alone or with various concentrations of tropisetron (TP: 0.3 to 10 mg/kg) at 30 min before APAP injection and were sacrificed 20 h after administration for assay of liver MPO. Results are mean ± SEM; *n* = 6 mice per group. ^#^ *P* < 0.005 versus control (normal saline); ^*∗*^ *P* < 0.05; ^*∗∗∗*^ *P* < 0.005 versus APAP alone.

Hepatic MPO activity is shown in Figure 2. A single dose of APAP (300 mg/kg) markedly elevated the hepatic MPO (*P* < 0.05) activity when compared with the control animals. Pretreatment with tropisetron 30 min prior to APAP administration attenuated hepatic MPO levels. Hepatic MPO levels were significantly decreased in tropisetron groups (1, 3, and 10 mg/kg) compared with the APAP-only group, respectively.

### 3.3. Effect of Tropisetron on GSH, SOD, and MDA Activities

**Figure 3:**
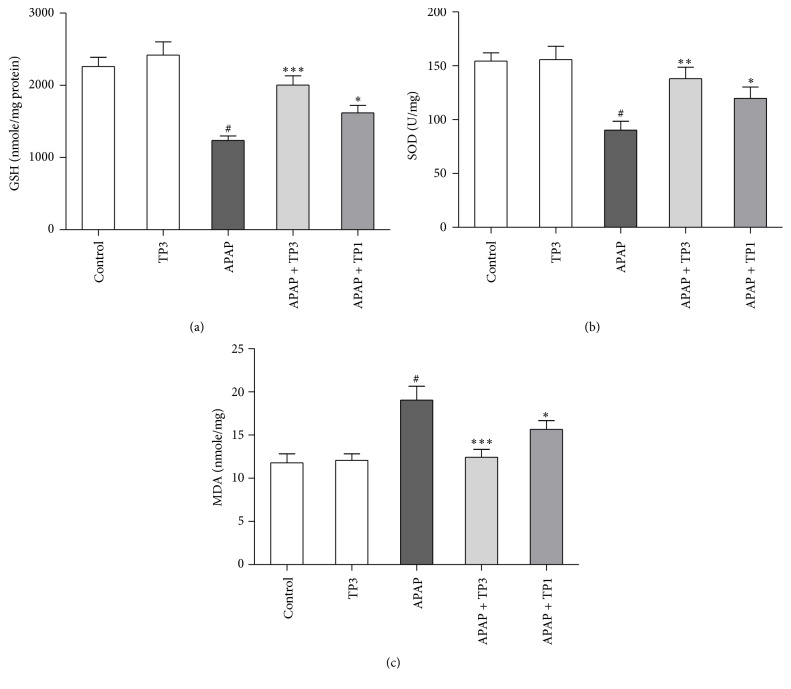
Tropisetron protects against APAP-induced hepatic GSH, SOD depletion, and MDA increase. Mice were administered with APAP 300 mg/kg alone or with tropisetron (TP: 1 and 3 mg/kg) at 30 min before APAP injection and were sacrificed 20 h after administration for assay of liver GSH (a), SOD (b), and MDA (c). Results are mean ± SEM; *n* = 6 mice per group. ^#^ *P* < 0.005 versus control (normal saline); ^*∗*^ *P* < 0.05; ^*∗∗*^ *P* < 0.01; ^*∗∗∗*^ *P* < 0.005 versus APAP alone.

Twenty hours after APAP administration, GSH and SOD concentrations were significantly reduced to 55% (*P* < 0.05) and 59% (*P* < 0.05) in the APAP group compared with the control animals. However, pretreatment with tropisetron 3 mg/kg significantly alleviated APAP-induced GSH depletion at 2005 ± 127 nmole/mg (*P* < 0.005) and SOD depletion at 138 ± 11 U/mg (*P* < 0.01). MDA levels were significantly increased by 162% in mice treated with APAP, indicating that APAP treatment significantly increased hepatic lipid peroxidation (*P* < 0.05). However, in mice administered with tropisetron (1 and 3 mg/kg) plus APAP, the MDA activity was markedly attenuated to 82% (*P* < 0.05) and 65% (*P* < 0.005) when compared with the APAP treated mice (Figure 3).

### 3.4. Effect of Tropisetron in Histology

**Figure 4:**
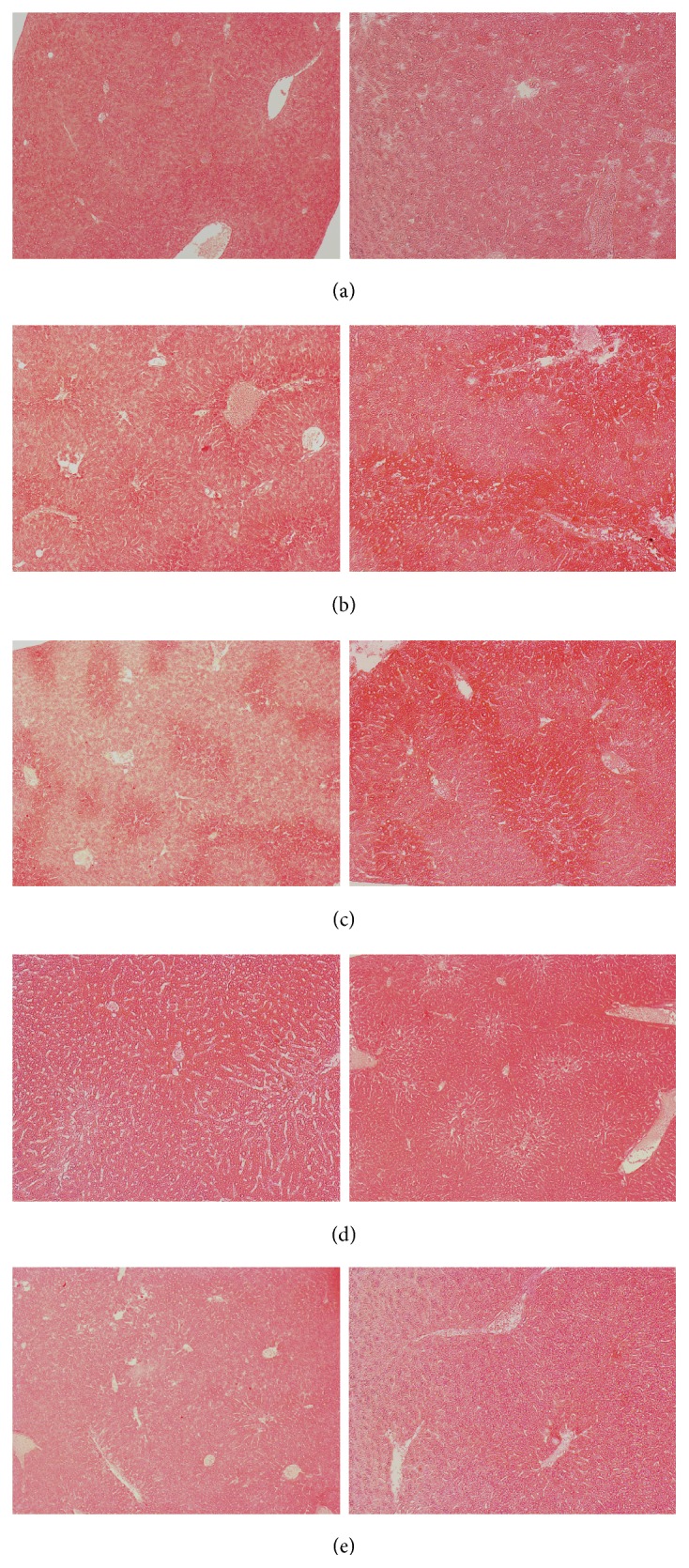
Tropisetron reduces APAP-induced liver injury in histology. Mice were given (a) control (normal saline) and (b) APAP 300 mg/kg alone or with various concentrations of tropisetron ((c) 1 mg/kg; (d) 3 mg/kg; (e) 10 mg/kg) at 30 min before APAP injection and were sacrificed 20 h after administration for assay by H&E stain (magnification: left column ×50, right column ×100).

As for histological analysis of tropisetron in APAP-induced liver injury (Figure 4), H&E staining demonstrated that APAP-only treated animal showed severe hepatic centrilobular necrosis and inflammatory cell infiltration, which were significantly reduced in the tropisetron plus APAP treated groups. Pretreatment of tropisetron significantly decreased hepatic inflammatory cell infiltration and diminished hepatocytes necrosis area.

### 3.5. Effect of Tropisetron on JNK/ERK MAPK Pathway

**Figure 5:**
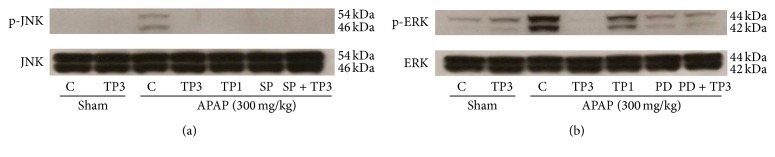
Tropisetron attenuates hepatic JNK and ERK expression in APAP-induced hepatic injury. (a) Hepatic p-JNK and JNK protein expression from shams receiving vehicle (sham + C: control; lane 1) or tropisetron (sham + TP3: 3 mg/kg; lane 2), treatment with acetaminophen (APAP: 300 mg/kg) receiving vehicle (APAP + control; lane 3), tropisetron (APAP + TP3: 3 mg/kg; lane 4), tropisetron (APAP + TP1: 1 mg/kg; lane 5), JNK inhibitor SP600125 (APAP + SP: 30 mg/kg; lane 6), or SP600125 plus tropisetron (APAP + SP + TP3; lane 7). (b) Hepatic p-ERK and ERK protein expression from shams receiving vehicle (sham + C: control; lane 1) or tropisetron (sham + TP3: 3 mg/kg; lane 2), treatment with acetaminophen (APAP: 300 mg/kg) receiving vehicle (APAP + control; lane 3), tropisetron (APAP + TP3: 3 mg/kg; lane 4), tropisetron (APAP + TP1: 1 mg/kg; lane 5), ERK1/2 inhibitor PD98059 (APAP + PD: 10 mg/kg; lane 6), or PD98059 plus tropisetron (APAP + PD + TP3; lane 7).

It has been reported that MAPK plays a key role in mediating APAP-induced hepatic intoxication in mice [20]. JNK activation is an important signal and mediates cell survival following toxicants-induced organ injury [21]. Therefore, we examined whether APAP-triggered JNK activation could be reduced by tropisetron administration. Our data showed that the levels of JNK and ERK phosphorylation markedly increased after APAP treatment (Figure 5). In treatment with different doses of tropisetron (1 and 3 mg/kg) 30 min before APAP administration, the expression of JNK and ERK phosphorylation decreased (Figures 5(a) and 5(b)). These results are consistent with our hypothesis that tropisetron suppresses the JNK/ERK MAPK signaling pathway.

### 3.6. Survival Outcome of Mice in Acetaminophen-Induced Liver Injury

We investigated whether tropisetron could prolong or improve the survival after APAP overdose; the twenty-four mice were randomly divided into three groups, including control (normal saline) group, APAP-treated (300 mg/kg) group, and APAP with tropisetron-pretreated (3 mg/kg) group. The 96 hours' survival rate was 100% in all experimental mice. In addition, there was not a statistical difference among the three groups in mice serum ALT levels at 96 hours.

## 4. Discussion

Tropisetron is a common antiemetic drug in clinical practice for prevention of nausea and vomiting [2, 25, 26]. A growing body of evidence also indicates that tropisetron has organ-protective function by attenuating inflammatory and apoptotic response [19–22]. However, little is known about the role of tropisetron in acetaminophen- (APAP-) induced hepatic damage. This study investigates the protective effects of tropisetron on acetaminophen-induced hepatotoxicity in mice. After acetaminophen-induced hepatic injury for 20 hours, plasma ALT concentration, hepatic MPO level, and MDA activity markedly increased, whereas GSH and SOD activity markedly decreased in mice. Pretreatment of tropisetron significantly improves these hepatic oxidant and damage parameters in mice subjected to acetaminophen-induced liver injury. Tropisetron pretreatment also prevented the acetaminophen-induced increase in hepatic phosphorylation of JNK and ERK. These results collectively suggest that the salutary effects of tropisetron seem to be correlated with the antioxidant activity and mediated via the JNK and ERK pathways on acetaminophen-induced hepatotoxicity in mice.

APAP is a common and safe antipyretic-analgesic medication when used at therapeutic doses [1, 27]. However, APAP overdose can induce hepatotoxicity, which may develop into liver failure [2, 3]. In our study, intraperitoneal administration of a single high dose of APAP 300 mg/kg markedly increased the severity of hepatic injury. The serum ALT level was markedly elevated compared with the normal control group. The significantly decreased serum ALT level in the tropisetron-administered groups prior to APAP showed its hepatoprotective effects. The histological analysis of liver section demonstrates a reduced centrilobular necrosis, fatty infiltration, and mononuclear cells infiltration in the tropisetron plus APAP treated mice with respect to the APAP-only treated mice (Figure 4). Previous studies have shown that when rodents were treated with APAP, there were infiltrations of mononuclear and polymorphonuclear cells in the centrilobular zones [10, 28]. It indicates that the inflammatory reaction was related to the release of chemotactic factors from the damage of hepatocytes [9, 29].

APAP is mainly metabolized* via* hepatic cytochrome P-450 to form an intermediate metabolite,* N*-acetyl-*p*-benzoquinone imine (NAPQI), which is primarily inactivated by conjugation with GSH. Excessive NAPQI depletes hepatic glutathione (GSH) and covalently binds to intracellular proteins, generating superoxide anion and free radicals resulting in oxidative stress reactions, mitochondria dysfunction, and hepatocyte damage [2, 3]. Therefore, hepatic GSH level is valuable indicator in protecting against APAP-induced hepatotoxicity, and enhancement of hepatic GSH is an important strategy for the treatment of APAP intoxication. To determine whether tropisetron could decrease APAP-induced GSH depletion, the hepatic GSH activity was measured. Our results showed that cotreatment with tropisetron and APAP decreased APAP-induced GSH depletion (Figure 3). The increased hepatic GSH level could in part explain the hepatoprotective mechanism of tropisetron. Previous evidences showed that while the GSH content decreased below a threshold level, hepatic xanthine dehydrogenase can be converted to reversible xanthine oxidase, having an influence on superoxide radical generation and production [30, 31]. SOD is thought to be an important antioxidant enzyme, for it is closely related to the elimination of reactive oxygen species. Therefore, the reduction of the activity of this enzyme may result in accumulation of superoxide radicals and hydrogen peroxide. The present study showed that APAP treatment causes depletion of the SOD antioxidant enzymes [32, 33]. Moreover, the enhanced SOD activities with hepatoprotective effects on the tropisetron plus APAP treated group are further supported (Figure 3). MDA is an important end-product of polyunsaturated fatty acids oxidative process, and tissue MDA level is an indicator of lipid peroxidation and oxidative stress [34, 35]. Our results showed that there was a higher hepatic MDA level in the APAP group in comparison with the control group. Related to the APAP group, the elevated hepatic MDA level decreased in the tropisetron plus APAP treated group.

JNK is an important regulator of mitochondrial permeabilization. It is well known that oxidative stress can directly activate the JNK pathway [12, 36]. Many studies have showed that oxidative stress caused JNK activation, either via redox alteration of the sequestration of JNK or through inhibition of JNK phosphatase [11, 12, 35]. APAP treatment induced activation of JNK is reflected in the increase of JNK phosphorylation in* in vitro* hepatocytes and* in vivo* livers [36, 37]. Activation of JNK was found to be important for oxidative stress-induced hepatocyte apoptosis. Studies have demonstrated that inhibition of JNK activity or suppression of JNK gene expression prevented hepatic damage from APAP overdose [38, 39]. Furthermore, JNK2 has been suggested to play a beneficial role in tissue repair after APAP overdose [12], and recent studies also showed that severity of APAP-induced liver injury is dependent on JNK activation and it may be a potential therapeutic target in APAP toxication [40, 41]. The ERK is similar to JNK in response to oxidative stress and is associated with cellular proliferation, survival, and differentiation [15, 37]. The ERK pathway plays an important role in APAP-induced liver injury, and the protective effect is usually accompanied by inhibition of activation of JNK/ERK [15, 42]. In this study, phospho-JNK and ERK expression significantly increased at 24 h after APAP treatment. Tropisetron pretreatment can effectively decrease phosphorylation of JNK1/2 and ERK1/2 (Figure 5). These results suggest that both ERK and JNK expressions promote the tropisetron protective effect in APAP-induced liver injury.

**Figure 6:**
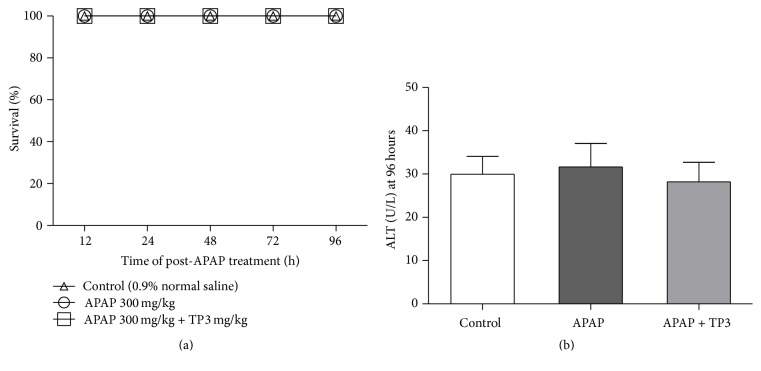
Survival analysis of APAP-induced liver injury in mice. Mice were intraperitoneally treated with 0.9% normal saline, with APAP 300 mg/kg alone, or with a 3 mg/kg concentration of tropisetron at 30 min before APAP injection. (a) The survival rate was measured until 96 h after normal saline or APAP 300 mg/kg administration (*n* = 8/each group). (b) All animals were sacrificed at 96 h after administration for assay of serum ALT.

Our results also showed that pre- or posttreatment with tropisetron (3 mg/kg) has similar protective effects in reducing APAP-induced liver injury (Figure 6). There is no significantly statistical difference in tropisetron-induced attenuation of liver injury between two groups. These findings suggested that tropisetron may have a therapeutic potential for preventing and treating APAP-induced liver injury. Previous evidence indicated that APAP overdose is induced over NAPQI binding cellular and mitochondrial proteins and formed protein adducts, which triggered toxic-metabolic reaction [2, 36]. NAPQI-adducts levels have been as the primary diagnostic marker of APAP intoxication and it is also an important indicator for evaluating drug therapeutic effect in APAP overdose [43]. Since NAPQI-adducts levels were not measured in our study, the effect of tropisetron on NAPQI-adduct expression in future study still needs to be clarified.

In conclusion, our data demonstrated that tropisetron can attenuate the APAP-induced hepatotoxicity in mice by enhancing the hepatic antioxidant activity and inhibiting the JNK/ERK MAPK activation and caspase-3 cleaves. However, further detailed studies are required to confirm its clinical application.

## Supplementary Material

To evaluate the effect of tropisetron in mice treated early after APAP overdose, we compared the pretreatment and posttreatment effect of tropisetron in APAP-induced liver. The results showed that pre- or posttreatment with tropisetron (3 mg/kg) has similar effects in reducing APAP-induced liver injury. However, there is no statistical difference between pretreated and posttreated groups with tropisetron (3 mg/kg).

## References

[1] Ji P., Wang Y., Li Z. (2012). Regulatory review of acetaminophen clinical pharmacology in young pediatric patients. *Journal of Pharmaceutical Sciences*.

[2] Lancaster E. M., Hiatt J. R., Zarrinpar A. (2014). Acetaminophen hepatotoxicity: an updated review. *Archives of Toxicology*.

[3] Hodgman M. J., Garrard A. R. (2012). A review of acetaminophen poisoning. *Critical Care Clinics*.

[4] Bajt M. L., Cover C., Lemasters J. J., Jaeschke H. (2006). Nuclear translocation of endonuclease G and apoptosis-inducing factor during acetaminophen-induced liver cell injury. *Toxicological Sciences*.

[5] Zarowitz B. J. (2012). Analgesic warfare: acetaminophen, really?. *Geriatric Nursing*.

[6] Zhang J.-Y., Song S.-D., Pang Q. (2015). Hydrogen-rich water protects against acetaminophen-induced hepatotoxicity in mice. *World Journal of Gastroenterology*.

[7] Ben-Ari Z., Mor E., Azarov D. (2005). Cathepsin B inactivation attenuates the apoptotic injury induced by ischemia/reperfusion of mouse liver. *Apoptosis*.

[8] Boulares A. H., Ren T. (2004). Mechanism of acetaminophen-induced apoptosis in cultured cells: roles of caspase-3, DNA fragmentation factor, and the Ca^2+^ and Mg^2+^ endonuclease DNAS1L3. *Basic & Clinical Pharmacology & Toxicology*.

[9] Liu Z.-X., Han D., Gunawan B., Kaplowitz N. (2006). Neutrophil depletion protects against murine acetaminophen hepatotoxicity. *Hepatology*.

[10] Williams C. D., Bajt M. L., Sharpe M. R., McGill M. R., Farhood A., Jaeschke H. (2014). Neutrophil activation during acetaminophen hepatotoxicity and repair in mice and humans. *Toxicology and Applied Pharmacology*.

[11] Latchoumycandane C., Goh C. W., Ong M. M. K., Boelsterli U. A. (2007). Mitochondrial protection by the JNK inhibitor leflunomide rescues mice from acetaminophen-induced liver injury. *Hepatology*.

[12] Bourdi M., Korrapati M. C., Chakraborty M., Yee S. B., Pohl L. R. (2008). Protective role of c-Jun N-terminal kinase 2 in acetaminophen-induced liver injury. *Biochemical and Biophysical Research Communications*.

[13] Saito C., Lemasters J. J., Jaeschke H. (2010). c-Jun N-terminal kinase modulates oxidant stress and peroxynitrite formation independent of inducible nitric oxide synthase in acetaminophen hepatotoxicity. *Toxicology and Applied Pharmacology*.

[14] Yu S.-M., Kim S.-J. (2015). The thymoquinone-induced production of reactive oxygen species promotes dedifferentiation through the ERK pathway and inflammation through the p38 and PI3K pathways in rabbit articular chondrocytes. *International Journal of Molecular Medicine*.

[15] Wang A.-Y., Lian L.-H., Jiang Y.-Z., Wu Y.-L., Nan J.-X. (2010). Gentiana manshurica Kitagawa prevents acetaminophen-induced acute hepatic injury in mice via inhibiting JNK/ERK MAPK pathway. *World Journal of Gastroenterology*.

[16] de la Vega L., Muñoz E., Calzado M. A. (2005). The 5-HT3 receptor antagonist tropisetron inhibits T cell activation by targeting the calcineurin pathway. *Biochemical Pharmacology*.

[17] Fiebich B. L., Akundi R. S., Lieb K. (2004). Antiinflammatory effects of 5-HT3 receptor antagonists in lipopolysaccharide-stimulated primary human monocytes. *Scandinavian Journal of Rheumatology*.

[18] Faerber L., Drechsler S., Ladenburger S., Gschaidmeier H., Fischer W. (2007). The neuronal 5-HT3 receptor network after 20 years of research-evolving concepts in management of pain and inflammation. *European Journal of Pharmacology*.

[19] Okamoto K., Kimura A., Donishi T. (2006). Contribution of peripheral 5-HT2A or 5-HT3 receptors to Fos expression in the trigeminal spinal nucleus produced by acute injury to the masseter muscle during persistent temporomandibular joint inflammation in rats. *Neuroscience*.

[20] Rahimian R., Fakhfouri G., Mehr S. E. (2013). Tropisetron attenuates amyloid-beta-induced inflammatory and apoptotic responses in rats. *European Journal of Clinical Investigation*.

[21] Mousavizadeh K., Rahimian R., Fakhfouri G., Aslani F. S., Ghafourifar P. (2009). Anti-inflammatory effects of 5-HT_3_ receptor antagonist, tropisetron on experimental colitis in rats. *European Journal of Clinical Investigation*.

[22] Zirak M. R., Rahimian R., Ghazi-Khansari M. (2014). Tropisetron attenuates cisplatin-induced nephrotoxicity in mice. *European Journal of Pharmacology*.

[23] Barzegar-Fallah A., Alimoradi H., Mehrzadi S. (2014). The neuroprotective effect of tropisetron on vincristine-induced neurotoxicity. *NeuroToxicology*.

[24] Liu F.-C., Yu H.-P., Hwang T.-L., Tsai Y.-F. (2012). Protective effect of tropisetron on rodent hepatic injury after trauma-hemorrhagic shock through P38 MAPK-dependent hemeoxygenase-1 expression. *PLoS ONE*.

[25] Ma Y., Su L., Liu L. (2015). Phase II clinical trial of palonosetron combined with tropisetron in preventing chemotherapy-induced nausea and vomiting. *International Journal of Clinical and Experimental Medicine*.

[26] Papadima A., Gourgiotis S., Lagoudianakis E. (2013). Granisetron versus tropisetron in the prevention of postoperative nausea and vomiting after total thyroidectomy. *Saudi Journal of Anaesthesia*.

[27] Blough E. R., Wu M. (2011). Acetaminophen: beyond pain and fever-relieving. *Frontiers in Pharmacology*.

[28] Wang X., Sun R., Chen Y., Lian Z.-X., Wei H., Tian Z. (2016). Regulatory T cells ameliorate acetaminophen-induced immune-mediated liver injury. *International Immunopharmacology*.

[29] Williams C. D., Bajt M. L., Farhood A., Jaeschke H. (2010). Acetaminophen-induced hepatic neutrophil accumulation and inflammatory liver injury in CD18-deficient mice. *Liver International*.

[30] Nýdlová E., Vrbová M., Česla P., Jankovičová B., Ventura K., Roušar T. (2014). Comparison of inhibitory effects between acetaminophen-glutathione conjugate and reduced glutathione in human glutathione reductase. *Journal of Applied Toxicology*.

[31] Saito C., Zwingmann C., Jaeschke H. (2010). Novel mechanisms of protection against acetaminophen hepatotoxicity in mice by glutathione and *N*-acetylcysteine. *Hepatology*.

[32] Agarwal R., MacMillan-Crow L. A., Rafferty T. M. (2011). Acetaminophen-induced hepatotoxicity in mice occurs with inhibition of activity and nitration of mitochondrial manganese superoxide dismutase. *Journal of Pharmacology and Experimental Therapeutics*.

[33] Kyle M. E., Miccadei S., Nakae D., Farber J. L. (1987). Superoxide dismutase and catalase protect cultured hepatocytes from the cytotoxicity of acetaminophen. *Biochemical and Biophysical Research Communications*.

[34] Cigremis Y., Turel H., Adiguzel K. (2009). The effects of acute acetaminophen toxicity on hepatic mRNA expression of SOD, CAT, GSH-Px, and levels of peroxynitrite, nitric oxide, reduced glutathione, and malondialdehyde in rabbit. *Molecular and Cellular Biochemistry*.

[35] Chen J., Zeng L., Xia T. (2015). Toward a biomarker of oxidative stress: a fluorescent probe for exogenous and endogenous malondialdehyde in living cells. *Analytical Chemistry*.

[36] Bhushan B., Borude P., Edwards G. (2013). Role of bile acids in liver injury and regeneration following acetaminophen overdose. *The American Journal of Pathology*.

[37] Conde de la Rosa L., Schoemaker M. H., Vrenken T. E. (2006). Superoxide anions and hydrogen peroxide induce hepatocyte death by different mechanisms: involvement of JNK and ERK MAP kinases. *Journal of Hepatology*.

[38] Gunawan B. K., Liu Z.-X., Han D., Hanawa N., Gaarde W. A., Kaplowitz N. (2006). c-Jun N-terminal kinase plays a major role in murine acetaminophen hepatotoxicity. *Gastroenterology*.

[39] Hanawa N., Shinohara M., Saberi B., Gaarde W. A., Han D., Kaplowitz N. (2008). Role of JNK translocation to mitochondria leading to inhibition of mitochondria bioenergetics in acetaminophen-induced liver injury. *The Journal of Biological Chemistry*.

[40] Du K., Xie Y., McGill M. R., Jaeschke H. (2015). Pathophysiological significance of c-jun N-terminal kinase in acetaminophen hepatotoxicity. *Expert Opinion on Drug Metabolism & Toxicology*.

[41] Du K., Williams C. D., McGill M. R., Jaeschke H. (2014). Lower susceptibility of female mice to acetaminophen hepatotoxicity: role of mitochondrial glutathione, oxidant stress and c-jun N-terminal kinase. *Toxicology and Applied Pharmacology*.

[42] Noh J.-R., Kim Y.-H., Hwang J. H. (2013). Davallialactone protects against acetaminophen overdose-induced liver injuries in mice. *Food and Chemical Toxicology*.

[43] McGill M. R., Lebofsky M., Norris H.-R. K. (2013). Plasma and liver acetaminophen-protein adduct levels in mice after acetaminophen treatment: dose-response, mechanisms, and clinical implications. *Toxicology and Applied Pharmacology*.

