# Supplementary figures and images for: Tropisetron Protects Against Acetaminophen-Induced Liver Injury via Suppressing Hepatic Oxidative Stress and Modulating the Activation of JNK/ERK MAPK Pathways
Source: Biomed Res Int. 2016 Nov 7;2016:1952947. doi: 10.1155/2016/1952947 (PMC5116490; doi:10.1155/2016/1952947)

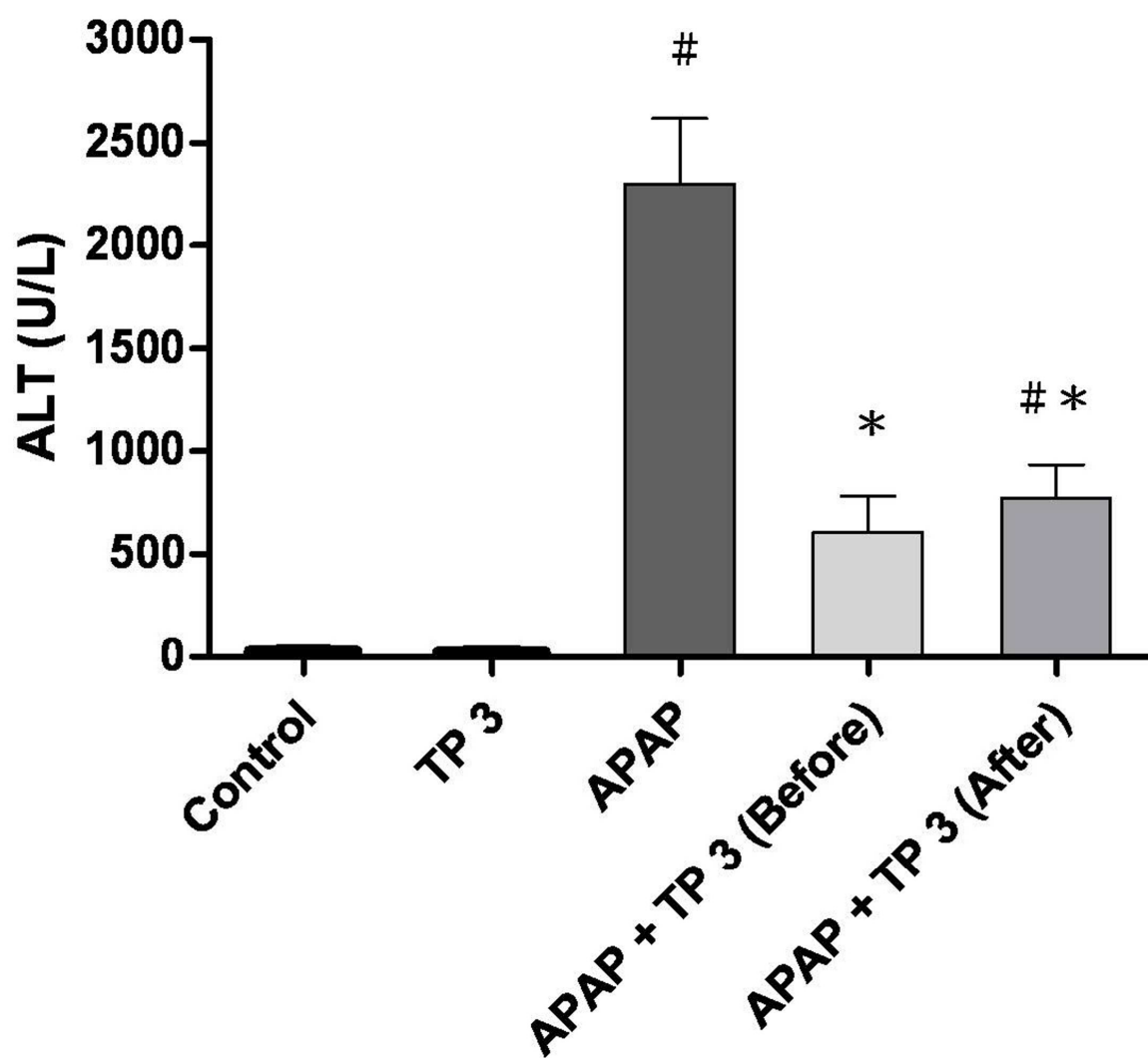

Fig. S1

Supplement: Supplementary file 1 — To evaluate the effect of tropisetron in mice treated early after APAP overdose, we compared the pretreatment and posttreatment effect of tropisetron in APAP-induced liver. The results showed that pre- or posttreatment with tropisetron (3 mg/kg) has similar effects in reducing APAP-induced liver injury. However, there is no statistical difference between pretreated and posttreated groups with tropisetron (3 mg/kg). [file 1952947.f1.pdf]
